# Development of New Deep Venous Thrombosis While on Apixaban

**DOI:** 10.1155/2017/2842935

**Published:** 2017-06-20

**Authors:** Munish Sharma, Sabarina Ramanathan, Koroush Khalighi

**Affiliations:** ^1^Department of Internal Medicine, Easton Hospital, Easton, PA, USA; ^2^Drexel University College of Medicine, Electrophysiology Department, Easton Hospital, Easton, PA, USA

## Abstract

The efficacy of novel oral anticoagulants (NOACs) in preventing deep venous thrombosis (DVT) has been established in large multicenter trials. Predictable pharmacokinetics, avoidance of routine laboratory monitoring, and lesser drug interactions have made NOACs safer and more tolerable treatment option in comparison to warfarin. However, cases of treatment failure mainly due to interindividual variation in plasma drug levels can be seen rarely. In this report we describe a case of acute DVT of right lower extremity in a patient who was on apixaban for prevention of venous thromboembolism (VTE) due to underlying nonvalvular atrial fibrillation (NVAF).

## 1. Introduction

NOACs including dabigatran, rivaroxaban, apixaban, and edoxaban are used for the prevention of stroke and peripheral thromboembolism in NVAF and for treatment and prophylaxis of VTE. Compared to vitamin K antagonists (VKAs) such as warfarin, NOACs have more predictable pharmacokinetics and do not need to be routinely monitored in lab for their plasma level. Their efficacy and safety relative to warfarin have been established in the major clinical trials [1–3]. However, due to the variation in the plasma drug level between the individuals, some patients may be at increased risk of treatment failure or bleeding events. Two of the major trials conducted on NOACs have also reported the wide variation in plasma drug level among the patients [4, 5]. We present a case of medication compliant patient who developed acute DVT of his right lower extremity in spite of being on apixaban 5 mg twice daily for VTE prophylaxis in the setting of persistent NVAF.

## 2. Case Report

On 4/7/2017, an 80-year-old male with history of recurrent/persistent symptomatic NVAF refractory to optimal medical therapy and multiple cardioversions, hypertension, and coronary artery disease (CAD) status after angioplasty with stent deployment to right coronary artery in 2001 underwent comprehensive electrophysiologic study, 3-dimensional mapping, intracardiac ultrasound, and AF ablation with pulmonary vein isolation. Vascular access was obtained through right femoral vein catheterization for the catheter ablation. He was on dabigatran 150 mg twice daily since the initial diagnosis of AF for thromboembolic prophylaxis but 3 months prior to this admission his anticoagulation was switched to apixaban 5 mg twice daily due to coverage issues with medical insurance. He was medication compliant. Apixaban was immediately resumed after AF ablation. Two days after the AF ablation, patient had ecchymosis and swelling of the right groin and an arterial Doppler study revealed a pseudoaneurysm arising from the common femoral artery measuring 3.5 × 2.1 × 2 cm. This was treated with thrombin injection. He was discharged home on metoprolol 100 mg twice daily, aspirin 81 mg daily, atorvastatin 40 mg daily, and apixaban 5 mg twice daily.

**Figure 1:**
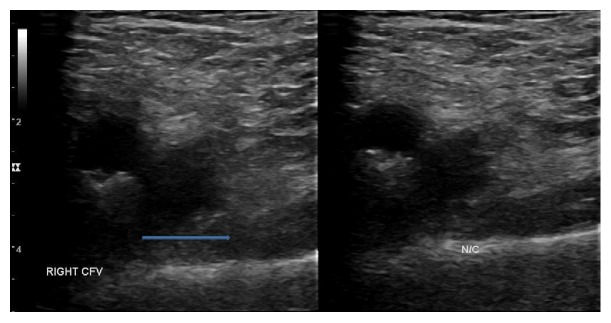
Noncompressibility of right common femoral vein (CFV) seen on duplex ultrasound.

**Figure 2:**
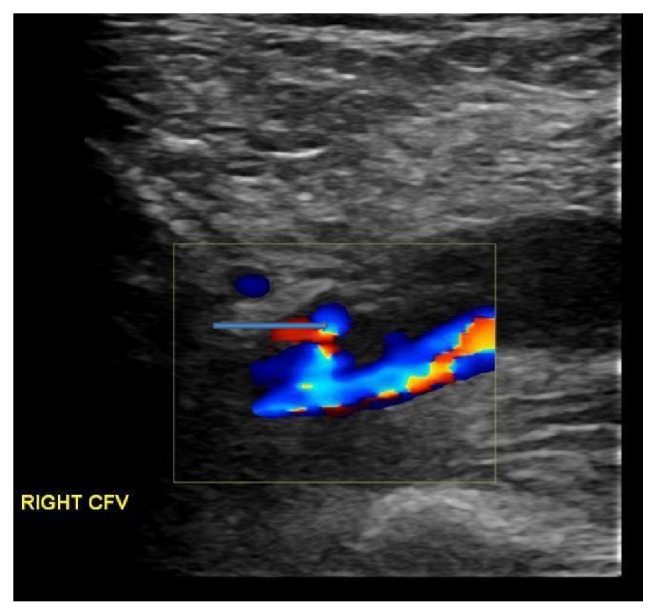
Turbulent flow seen distal to obstruction at right common femoral vein (CFV).

On 4/18/2017, patient was readmitted to the hospital because of new onset swelling and redness of right lower extremity that started 5 days after previous hospital discharge. A venous Doppler of the right lower extremity showed acute nonocclusive DVT in the common femoral and the distal external iliac vein (Figures 1 and 2). On examination, vitals were stable with normal sinus rhythm. There was no carotid bruit. Cardiac examination revealed normal S1 and S2, regular at a rate of 70 bpm with no evidence of murmur/rub or gallop. Right lower extremity showed pitting pedal edema extending up to the mid shin level. There was no worsening of the hematoma in the right groin region. International normalized ratio (INR), activated partial thromboplastin time (aPTT), and platelets and fibrinogen level were normal. Anti-factor Xa was not measured. In view of new onset DVT, apixaban was discontinued and patient was started on a heparin drip bridged with warfarin with a target international normalized ratio (INR) of 2-3. His other home medications were continued with the plan to follow up as an outpatient.

## 3. Discussion

VTE has an annual incidence of 1 to 2 cases per 1000 people in the general population [6]. Conventional treatment consists of a parenteral anticoagulant, such as enoxaparin, for at least 5 days, and warfarin begun during this time and continued for at least 3 months. Recently, the use of warfarin for treatment and prevention of pulmonary embolism, DVT, and AF has significantly declined with increasing preference for NOACs. Need for frequent monitoring of individualized dosing to maintain INR within a narrow therapeutic range, need for bridging therapy with parenteral anticoagulants, and significant risk of bleeding events have been obviated with advent of NOACs. Warfarin has been found to have twice as many major bleeding episodes compared to placebo with a target INR between 2.0 and 3.0 [7, 8]. Conversely, predictable pharmacokinetics, exclusion of laboratory monitoring of the plasma drug level, lesser drug interactions, and fewer chances of adverse event such as bleeding episodes have made NOACs safer and more tolerable treatment option. Apixaban is one such NOAC, which acts by inhibiting the cleavage of prothrombin into thrombin during the final step of coagulation cascade. Apixaban for the Initial Management of Pulmonary Embolism and Deep-Vein Thrombosis as First-Line Therapy (AMPLIFY) trial showed noninferiority when compared with warfarin in the treatment and prophylaxis of VTE with a significant reduction in bleeding events. It also showed very low recurrence rate of 0.68% by day 7 of initiation of treatment indicating that the early recurrence of thromboembolic event is uncommon with apixaban [9]. With emerging reports of variability in the efficacy and safety of the NOACs among certain patient subgroups [10], question of tailoring therapy by means of plasma drug levels has started to linger in the mind of many. Though studies on some of the NOACs have recommended to personalize the dose based on patient characteristics such as age, sex, and creatinine clearance, there are certain circumstances in which the direct quantification of a NOACs plasma level is thought to be more informative [11]. Liquid chromatography tandem mass spectrometry can be used to directly measure plasma drug concentration of NOACs but they are not widely available. Also, standard assays such as PT/INR level vary greatly depending on the reagent and analyzer used. There are certain systematic reviews of laboratory measurements of NOACs that have been published recently. Among them, anti-factor Xa activity has been considered sensitive throughout therapeutic range when calibrated to apixaban. Normal anti-FXa level excludes significant drug levels. The limited availability of this test makes it difficult to be routinely used [12, 13].

In our case, patient was compliant with his prescribed apixaban dose, there was no significant delay in resumption of apixaban after catheter ablation of AF, and he had no history of coagulation disorder in himself or family member and did not have previous history of VTE. Patient did not receive any medication at the time of development of DVT that is known to reduce the efficacy of apixaban. In the setting of possible anticoagulation failure, decision to switch to a different agent can be difficult especially in cases of NOACs, since dosing is not based on laboratory measurements that would confirm the adequate anticoagulation. There is no definite data suggesting the switching of one NOAC to another and we decided to opt for a more conventional agent like warfarin with a readily available monitoring tool for plasma drug level.

## 4. Conclusion

Cases of treatment failure with NOACs are concerning for patient safety and emphasize the need for more reliable assays reflecting NOAC activity since the standard assays of anticoagulation are generally insufficient. In this scenario, conventional agent like warfarin with accurate monitoring tools for serum drug level can be a suitable alternative.
